# A New Treatment Option in Incomplete Partition Type III: The Varese Bone–Air Stimulation (B.A.S.)

**DOI:** 10.3390/jpm13040681

**Published:** 2023-04-19

**Authors:** Flavia Di Maro, Vittoria Sykopetrites, Annalisa Meli, Davide Cocozza, Greta Albanese, Maria Teresa Antonietta Miccoli, Annalisa De Candia, Mario Picozzi, Francesca Greco, Eliana Cristofari

**Affiliations:** 1 Audiovestibology Unit Varese Hospital, 21100 Varese, Italy; 2 Center for Clinical Ethics, Biotechnology and Science of Life Department, Insubria University, 21100 Varese, Italy

**Keywords:** incomplete partition type III, X-linked deafness, bone–air stimulation

## Abstract

The incomplete partition type III is a severe cochlear malformation present in X-linked deafness. It is a rare, non-syndromic cause of severe to profound mixed hearing loss, often progressive. The complete absence of bony modiolus and the wide communication between the cochlea and the internal auditory canal make cochlear implantation challenging, with still no consensus on the management of these patients. To the best of our knowledge, no results have ever been published in the literature on the treatment of these patients with hybrid stimulation (bone and air). We present three cases in which this hybrid stimulation gave better audiological results then air stimulation alone. A literature review on audiological results of the current treatment options in children affected by IPIII malformation was conducted independently by two researchers. Ethical considerations on the treatment of these patients were conducted by the Bioethics department of the University of Insubria. In two of the patients, the bone–air stimulation, associated with prosthetic–cognitive rehabilitation, meant that surgery was avoided, obtaining similar communication performances of those present in the literature. We believe that, when the bone threshold appears partially preserved, a stimulation through the bone or hybrid modality, such as the Varese B.A.S. stimulation, should be attempted.

## 1. Introduction

X-linked deafness is a rare condition caused by mutations in POU3F4, a gene related to brain and cochlear development. The mutation results in a severe cochlear malformation classified as incomplete partition type III (IPIII), which consists of an absent bony modiolus and no separation between the base of the cochlea and the Internal Auditory Canal (IAC). The bony interscalar septa and the external dimension of the cochlea are preserved [1]. Males present with severe to profound mixed hearing loss, often progressive, whereas females usually have a lower degree of audiological impairment. The IPIII represents one of the most challenging cochlear malformations to manage surgically, with still no consensus on the management of these patients, also considering the uncertain outcome.

In the case of moderate to severe hearing loss, a traditional hearing aid may be sufficient to ensure adequate communication, however the majority of IPIII patients present with severe to profound mixed hearing loss [2,3]. The conductive component of the hearing loss is assumed to be caused by stapes fixation [1] but there has been speculation on other theories, such as a rise in the perilymphatic pressure [3] or the presence of a third window phenomenon [4]. There are no results reported in the literature of patients treated with bone stimulation, presumably because the bone thresholds were considered too low for bone conduction hearing [5].

Cochlear implantation is possible in IPIII, albeit burdened with significant complications, especially due to the wide communication between the cochlea and the IAC. A severe gusher is practically unavoidable, with a combined difficulty in performing a correct placement of the array in a malformed cavity, and an increased risk of meningitis due to the ample cerebrospinal fluid (CSF) leak or the necessity of a lumbar drainage [1,6]. Moreover, the risk of array displacement in the IAC is high and even if it is correctly placed, it can migrate over time [6,7].

On the other hand, the worsening in the auditory performances over time could destine the child to a life of sound deprivation.

Bone conduction stimulation is an alternative to air stimulation that propagates sound directly to the inner ear through skull bone vibrations. In patients with a preserved hearing bone threshold, this method can be utilized to restore hearing discrimination even if the air threshold is compromised. This is possible using bone conduction hearing devices that do not always necessitate surgery, which would be a simple procedure. Patients with IPIII malformation and severe to profound mixed hearing loss are nowadays given the only treatment option of cochlear implantation.

To the best of our knowledge, no results have ever been published in the literature on the treatment of these patients with hybrid stimulation (bone and air).

We present three cases of children with IPIII malformation treated with hybrid bone–air stimulation (B.A.S.) and the Varese B.A.S. audiological features and results are carefully described. A literature review was conducted on the clinical and audiological outcomes of all the known treatments available for patients with IPIII malformation. In light of the data that emerged, an analysis of the ethical implications of the therapeutic options available for these patients was performed, with consideration of the risks and potential benefits of each intervention.

## 2. Materials and Methods

A retrospective review of all pediatric patients with IPIII malformation treated in the referral center SSD of Audiovestibology Varese from 2001 to 2022 was conducted. Inclusion criteria were: the presence of mixed hearing loss, a bone hearing threshold of at least 60 dB HL and the radiological evidence of IPIII malformation.

Audiological data (Pure Tone Audiometry, Categories of Auditory Performance, Speech Reception Thresholds (SRTs) in noise), anamnestic information and follow-up data were collected. The audiological results obtained with air, bone and hybrid stimulation were statistically analyzed. The graphical representation and the statistical analysis were performed using Student’s *t*-test with GraphPad Software (GraphPad Software version 9.5.1, Boston, USA) with a significance value of *p* < 0.05. Informed consent was obtained from all subjects involved in the study.

### 2.1. Device Settings

Both devices using either the airway or bone route are acceptable amplification options for patients with conductive or mixed hearing loss, whereas behind the ear hearing aids (BTEs) are usually the first choice for patients with sensorineural hearing loss. In the Varese B.A.S., a BTE hearing aid and a bone conduction device are employed simultaneously in each patient. A powerful BTE hearing aid was used to treat the aerial component of hearing loss, namely Phonak^®^ SKY Ultra Power. The devices were adjusted according to the patient’s audiometric threshold. The DSL^®^ 5 pediatric prescription formula, with adaptive microphones and feedback suppression systems, was used as an amplification target. The target gain subjectively did not guarantee sufficient loudness, so it was increased based on the patient’s responses. A bone conduction device coupled with a soft band was used to stimulate the bone component. To guarantee the best gain and maximum power output, the BAHA^®^ 5 Superpower was chosen: which, in addition to being one of the most powerful bone conduction devices, is the only one equipped with the EveryWear™ standard. The transducer was placed on the contralateral mastoid through a connecting cable. The BAHA^®^ was adjusted based on the audiometric threshold detected through the worn device (so-called bone conduction direct). The proprietary cochlear BAHA prescription formula was used. The daily program was set as follows: microphone set in adaptive mode, active feedback management (after critical gain measurement) and position compensation set to “on”. In addition, the gain was increased to satisfy the loudness required by the patient. Despite the critical gain measurement, the new volume increase required a review of the feedback.

### 2.2. Test Procedure

Speech reception thresholds (SRTs) in noise were measured using an adaptive testing procedure, the simplified Italian matrix test (SiIMax). The simplified version uses a shortened version of the original word matrix, forming a three-word sentence with an unpredictable meaning, and is specially designed for pediatric patients or adults with a reduced word span. Measurements were conducted during the follow-up visits in several sessions which allowed the patient to become familiar with the test. All measurements were conducted in a silent booth with the patient one meter away from a single loudspeaker. The signal was kept constant at 65 dB SPL and the noise varied from an initial signal to noise ratio (SNR) of 0 from the same front source. Both OlSa-noise and the International Speech Test Signal (ISTS), with different masking modulation effects, were used as competition to the signal. The participants conducted the tests with the various configurations of the devices as shown later. In our department, the highest accepted value was +20 S/R, beyond this limit the results were considered not significant.

A literature review on audiological results of the current treatment options in children affected by IPIII malformation was conducted independently by two researchers. The results were analyzed by the Bioethics department of the University of Insubria and ethical considerations were conducted on the treatment of these patients.

## 3. Results

Among 1004 pediatric patients followed because of hearing loss or with risk factors for hearing loss in the Audiovestibology Unit of Varese in 2022, five patients had the radiological diagnosis of IPIII malformation. All of the patients had mixed hearing loss. Two patients were excluded from the study due to a bone threshold above the value of 60 dB HL.

**Figure 1:**
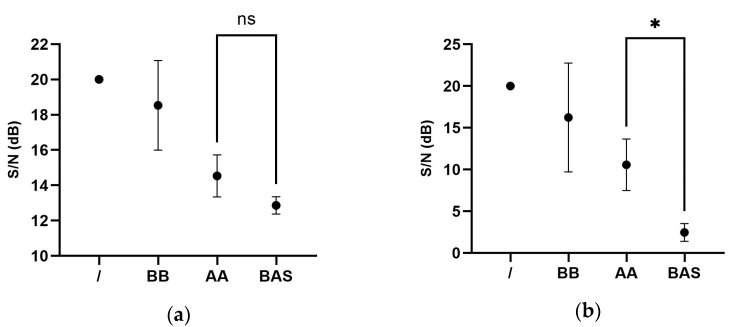
Statistical analysis of the audiological result on simplified Italian matrix test (SiIMax) with different competition signals. (**a**) International Speech Test Signal (ISTS) (**b**) OlSa-Noise. B.A.S. _ = Bone–Air Stimulation; “/” = no stimulation; BB = bilateral bone stimulation; AA = bilateral air stimulation; B.A.S. = Hybrid Bone–Air stimulation on the best configuration; “*” statistically significant; “ns” not statistically significant.

Three patients were included in the study and their cases are shown below. Audiological results are shown in Table 1. The statistical analysis of the audiological results is shown in Figure 1. The B.A.S. stimulation resulted in better audiological results using both OlSa-noise and International Speech Test Signal (ISTS) signals, but only in the second condition was the difference statistically significant (*p* = 0.0126).

### 3.1. CASE 1

#### N.M. 12 y.o. Male

He was born preterm (35 w) from a pregnancy complicated by maternal hypothyroidism. The birth weight was 3100 g with Apgar 8–10. He presented with neonatal jaundice (bilirubin 21 mg/dL) treated with prolonged phototherapy (10 days). The outcome of the neonatal auditory screening was not available. The mother had undergone stapes surgery in another department for mixed hearing loss with an intraoperative finding of a severe gusher which required the suspension of the surgery.

The first evaluation of the child at our Audiovestibology Unit was made at the age of 1 year with diagnosis of moderate to severe bilateral mixed hearing loss requiring a hearing aid and adequate speech therapy training. He underwent CT (Computed Tomography) scan and MRI (Magnetic Resonance Imaging) with a finding of bilateral IPIII malformation.

The child grew up following our prosthetic-rehabilitation program achieving the normal stages of language development. The hearing threshold worsened, up to the age of five y.o. when the air prosthesis was no longer adequate, and it was indicated he should receive cochlear implantation. A severe gusher occurred during surgery and the procedure, even with the agreement of the parents, was suspended. Fortunately, the threshold was preserved, and the child continued to use his hearing aid. The parents have always refused further attempts to place a cochlear implant.

At school, the child used a wireless remote microphone with good academic performances, until the age of nine, when the child started complaining of difficulty in understanding the teacher and a deflated mood. Although the air thresholds were low, the bone thresholds were partially preserved and to the suggestion of a BAHA softband in association with his air stimulation was made. Audiological results are shown in Table 1.

After three years of follow-up, the child presents with slightly worsened left air and bone thresholds, however, he continues to constantly wear the double prosthesis with benefit with acceptable communicative and scholastic results. Communication was evaluated on the Categories of Auditory Performance (CAP) scale (Score = 4) and he reached score 5 according to the Speech Intelligibility Rating (SIR).

### 3.2. CASE 2

#### S.S. 9 y.o. Male

He was full-term born from a regular pregnancy. There were no maternal infections and the birth weight was 3170 g. There were no perinatal complications. He had no family history of hearing loss. He was referred to our department at the age of 13 months, after failing the newborn auditory screening. He was diagnosed with moderate to severe bilateral sensorineural hearing loss and inadequate communicative performances. It was suggested that the child received an immediate hearing aid fitting and speech rehabilitation and then he underwent a CT scan and MRI with a finding of bilateral IPIII malformation. The child was later diagnosed with mild cognitive retardation; however, he attended school with a support teacher and benefited from wireless remote microphone. At the age of 6 y.o., the hearing thresholds worsened to a profound mixed hearing loss and, with a partially preserved bone threshold, a BAHA superpower softband was prescribed in addition to his air stimulation. Audiological results are shown in Table 1. The child showed subsequent immediate benefit and after 3 years of follow-up, still wears and benefits from both the aids (bone stimulation on the right side and air stimulation on the left side) with good scholastic results. In these three years, the air threshold has slightly worsened but the bone threshold remains stable without affecting the communication performances. His score at the CAP scale was 5 and he reached score 5 according to the SIR test.

### 3.3. CASE 3

#### D.M. 9 y.o. Male

He was full-term born from a regular pregnancy. There were no known maternal infections during pregnancy. The birth weight was 3000 g with Apgar 9–10. No perinatal complications occurred. He had no family history of deafness. He presented with language delay. The parents’ first doubts about hearing emerged at the age of two. The diagnosis of deafness was performed in another department with the first use of a hearing aid at the age of four with unsatisfactory communication results. He underwent a CT scan and MRI with a finding of bilateral IPIII malformation. A cochlear implant was proposed which the parents refused. At the age of 5 y.o., the patient was brought to our department, with a diagnosis of mild to severe bilateral mixed hearing loss with indications for a prosthetic upgrade, rehabilitation management and use of a wireless remote microphone. After the initial benefit, the thresholds gradually worsened and the possibility of performing a cochlear implant was re-evaluated at the age of 8. Given the high surgical risks, in agreement with the parents, a bone conduction device coupled with a soft band was fitted in association with his air stimulation with a consistent benefit from the double stimulation. The audiological results are shown in Table 1. Despite the improved results with the B.A.S. stimulation, the patient refused the bone conduction device and maintained a bilateral air stimulation.

**Figure 2:**
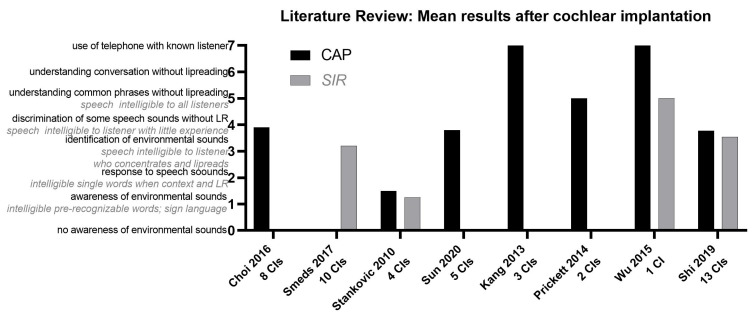
Literature review: mean results after cochlear implantation [5,6,10,15,17,19,20,21]. CAP (black): categories of auditory performance scale; SIR (grey italics): speech intelligibility rating test; LR: lip reading; CI: cochlear implant.

A total of 178 abstracts published after 2009 were selected on the basis of the following keywords “IP III malformation”, “X-Linked deafness”. Titles and abstract were screened and 29 articles were fully reviewed. The final analysis was conducted on 17 papers [5,6,8,9,10,11,12,13,14,15,16,17,18,19,20,21,22] due the lack of audiological data described in the other articles. However, the audiological evaluation varied greatly between different authors which prevented a direct comparison between the results. A graphical representation of the tests most represented in the various studies, Categories of Auditory Performance (CAP) scale and Speech Intelligibility Rating (SIR), is illustrated in Figure 2.

## 4. Discussion

Incomplete partition type III is a severe cochlear malformation with an estimated incidence of 0.9–2% of all inner ear malformations [1,23]. In our cohort of children followed up for hearing loss or with risk factors for hearing loss in 2022, the estimated prevalence was 0.5%.

Traditionally, these patients are treated with hearing aids until the progressive worsening of the acoustic threshold leads to cochlear implantation, an operation burdened by high surgical risks, especially when performed on very young children.

Moreover, the literature outcomes of cochlear implantation in IPIII malformation are very discordant, when and if adequately described. The audiological tests performed are different and often not comparable.

Smeds et al. [5] describe the audiological outcome of 10 patients with IPIII malformation who underwent 15 cochlear implantations at an average age of 1.8 years (0.9–2.8 yo). In four patients, the array was displaced in the IAC with intraoperative repositioning. Four surgeries resulted in a partial insertion and one patient presented with a post-operative CSF leak, managed conservatively. Audiological results showed the achievement of sound detection in three cases, in six patients a mean word score of 48% in quiet and, in five patients, 24% in noise. Despite the fact that the preoperative pure-tone average bone-conduction threshold ranged from 28 to 65 dB HL, only one child was tested with a bone-anchored hearing device before cochlear implantation. The threshold of all the cases was considered too poor for useful bone stimulation with available devices. The authors expressed the hope that the advent of more powerful bone-anchored devices could represent a therapeutic alternative for patients with analogous characteristics.

Other authors reported the audiological outcomes varying from sound detection to a CNC word recognition score of 100% passing through a series of different audiological tests that make it difficult to compare the results of the various studies [6,7,8,9,10,11,12,13,14,15,16,17,18,19,20,21,22].

The most used tool to measure the CI-related discrimination abilities in the literature, of patients with IPIII, are the CAP (Categories of Auditory Performance) and the SIR (Speech Intelligibility Rating) scales. On average, the results reveal recognition of some speech sounds without lip reading. However, the rehabilitation method used is never described.

According to the aforementioned tests, our patients benefited from hybrid bone and air stimulation and they achieved speech production equal to those of normal hearing children of the same age.

However, the audiological result alone is not enough to justify the communication results of these patients. Once the acoustic perception is guaranteed, all patients have to undergo a period of rehabilitation aimed at developing communication abilities. In our department, all deaf patients, regardless of the etiology of deafness, undergo an initial prosthetic-rehabilitation method, as described in other articles [24]. A trans-disciplinary rehabilitation method should involve the figures of the medical doctor specialized in Ear and Hearing diseases, the speech therapist, the pedagogist, audiometrist and audioprosthetist in order to achieve the following main objectives:

- early and correct audiological diagnosis;
- prosthetic–cognitive rehabilitation in order to correct the sensory impairment and the consequent communicative handicap. With this kind of rehabilitation, described by S. Burdo [25,26], it is possible to maximize the cognitive outcome of patients taking full advantages of the most recent audiological technology.

In our experience, with the limits of the cognitive variables peculiar to each case, as well as the family adherence to the rehabilitative method, the residual cerebral plasticity and the auditory memory at the time of diagnosis, patients are given the opportunity to achieve a complete scholastic integration with comparable communicative performances to those of normal hearing.

### Ethical Considerations

Criteria for ethical issues concerning patients being treated with B.A.S. involve various aspects.

Decision-making in children is a very complex problem because there are essentially two apparently conflicting requirements. The first is related to the need to perform the effective intervention in time to guarantee the recovery of perceptive and linguistic abilities; the second is related to the need to avoid creating functional damage to the child. Parents have the burden of a choice which on the one hand contemplates a surgical intervention with high intraoperative risks and no guarantees of success and on the other an acoustic stimulation which allows the maintenance of effective communication performances associated with a close audiological and pedagogical follow-up.

Parents not only have an accountability to ensure an acceptable quality of life for their child, but also to promote the development of their future autonomy and self-fulfillment.

From a careful analysis of the audiological results published in the literature and those achieved by the patients involved in the study, we believe that the application of double air and bone acoustic stimulation is proportionate [27] and ethically acceptable as it allows the maintenance of good communication performances, which is the first goal to be achieved in the treatment of every deaf child, assuring him/her an open future [28].

If any critical audiological and pedagogical issues are identified during the follow-up, the most appropriate therapeutic procedure is re-discussed with the parents.

## 5. Conclusions

IPIII malformation is a severe condition related to hearing loss that often makes pediatric patients eligible for cochlear implantation. When the bone thresholds are partially preserved, a stimulation through the bone or hybrid modality, such as the Varese B.A.S. stimulation, should be attempted. In our experience, this type of stimulation, associated with a correct rehabilitation, can avoid subjecting young children to a surgery burdened by potential serious complications, and to obtain similar communication performances at the same time. However, further studies with a larger sample size are needed to confirm the statements reported.

## Figures and Tables

**Table 1:** Audiological results with Bone, Air and Hybrid (B.A.S.) stimulation in various configurations. BIN = binaural. PTA = Pure Tone Average. AC = Air Conduction. BC = Bone Conduction.

Name	Age (Years)	PTA (0.5–1–2–4 KHz) AC Right	PTA (0.5–1–2–4 KHz) AC Left	PTA (0.5–1–2–4 KHz) BC Right	PTA (0.5–1–2–4 KHz) BC Left	Matrix Test: Noise	Matrix Test: ISTS
N.M. (Case 1)	12	96.25	105	51.25	51.25	BONE BIN: >+20 AIR right: +11.6 B.A.S.-Air right and Bone left: +1.3	BONE BIN: +20 AIR right: +13.7 B.A.S.-Air right and Bone left: +13.2
S.S. (Case 2)	9	103.75	106.25	50	52.5	BONE BIN: +8.7 AIR BIN: +7.1 B.A.S.-Bone right and Air left: +2.7 B.A.S.-Bone left and Air right: +5.6	BONE BIN: +15.6 AIR BIN: +15.9 B.A.S.-Bone right and Air left: +13.1 B.A.S.-Bone left and Air right: +14.6
D.M. (Case 3)	9	82.5	91.25	58.75	58.75	BONE BIN: +20 AIR BIN: +13 B.A.S.-Bone right and Air left: +3.4 B.A.S.-Bone left and Air right: +20	BONE BIN: +20 AIR BIN: +14 B.A.S.-Bone right and Air left: +12.3 B.A.S.-Bone left and Air right: +18

## Data Availability

The data presented in this study are available on request from the corresponding author. The data are not publicly available due to privacy issues.
